# Use of Autologous Cord Blood Mononuclear Cells Infusion for the Prevention of Bronchopulmonary Dysplasia in Extremely Preterm Neonates: A Study Protocol for a Placebo-Controlled Randomized Multicenter Trial [NCT04440670]

**DOI:** 10.3389/fped.2020.00136

**Published:** 2020-04-02

**Authors:** Zhuxiao Ren, Xu Fang, Qi Zhang, Y. G. Mai, X. Y. Tang, Q. Q. Wang, C. H. Lai, W. H. Mo, Y. H. Dai, Q. Meng, Jing Wu, Z. Z. Ao, H. Q. Jiang, Yong Yang, L. H. Qu, C. B. Deng, Wei Wei, Yongsheng Li, QI Wang, Jie Yang

**Affiliations:** ^1^Department of Neonatology, School of Medicine, Jinan University, Guangzhou, China; ^2^Department of Neonatology, Guangdong Women and Children Hospital, Guangzhou Medical University, Guangzhou, China; ^3^Department of Neonatology, Sun Yat-sen Memorial Hospital, Sun Yat-sen University, Guangzhou, China; ^4^Department of Neonatology, The Third Affiliated Hospital of Sun Yat-sen University, Guangzhou, China; ^5^Department of Neonatology, Nanfang Hospital, Southern Medical University, Guangzhou, China; ^6^Department of Neonatology, Zhongshan Boai Hospital, Zhongshan, China; ^7^Department of Neonatology, Foshan Chancheng Central Hospital, Foshan, China; ^8^Department of Neonatology, Foshan Women and Children Hospital, Foshan, China; ^9^Department of Neonatology, Guangdong Second Provincial General Hospital, Guangzhou, China; ^10^Department of Neonatology, Hexian Memorial Affiliated Hospital of Southern Medical University, Guangzhou, China; ^11^Department of Neonatology, Heyuan Women and Children Hospital, Heyuan, China; ^12^Department of Neonatology, Jiangmen Women and Children Hospital Jiangmen, China; ^13^Department of Neonatology, Dongguan Women and Children Hospital, Dongguan, China; ^14^Department of Neonatology, Guangzhou Huadu Women and Children Hospital, Guangzhou, China; ^15^Department of Neonatology, The Fifth Affiliated Hospital of Guangzhou Medical University, Guangzhou, China; ^16^Guang Dong Cord Blood and Stem Cell Bank, Guangzhou, China

**Keywords:** cord blood cells, bronchopulmonary dysplasia, prevention, extremely preterm infants, autologous

## Abstract

**Background:** Despite the rapid advance of neonatal care, bronchopulmonary dysplasia (BPD) remains a significant burden for the preterm population, and there is a lack of effective intervention. Stem cell depletion because of preterm birth is regarded as one of the underlying pathological mechanisms for the arrest of alveolar and vascular development. Preclinical and small-sample clinical studies have proven the efficacy and safety of stem cells in treating and preventing lung injury. However, there are currently no randomized clinical trials (RCTs) investigating the use of autologous cord blood mononuclear cells (ACBMNC) for the prevention of BPD in premature infants. The purpose of this study is to investigate the effects of infusion of ACBMNC for the prevention of BPD in preterm neonates <28 weeks.

**Methods:** In this prospective, randomized controlled double-blind multi-center clinical trial, 200 preterm neonates <28 weeks gestation will be randomly assigned to receive intravenous ACBMNC infusion (5 × 10^7^ cells/kg) or placebo (normal saline) within 24 h after birth in a 1:1 ratio using a central randomization system. The primary outcome will be survival without BPD at 36 weeks of postmenstrual age or at discharge, whichever comes first. The secondary outcomes will include the mortality rate, other common preterm complication rates, respiratory support duration, length, and cost of hospitalization, and long-term outcomes after a 2-year follow-up.

**Conclusion:** This will be the first randomized, controlled, blinded trial to evaluate the efficacy of ACBMNC infusion as a prevention therapy for BPD. The results of this trial will provide valuable clinical evidence for recommendations on the management of BPD in extremely preterm infants.

**Clinical Trial Registration:** ClinicalTrials.gov, NCT04440670, registered 06/18/2020, prospectively registered, https://clinicaltrials.gov/study/NCT04440670?term=NCT04440670&rank=1 (Additional File 2).

## Background

Preterm birth, a significant growing health concern around the world, affects 5–18% of newborn infants (1, 2). Bronchopulmonary dysplasia (BPD) is a severe and frustrating preterm complication causing adverse long-term outcomes (3, 4). The incidence of BPD in extremely preterm neonates was reported to be as high as 68% (5). The disruption of normal pulmonary vascular and alveolar growth after early birth subjects these infants to increased cardiopulmonary morbidity and mortality (6, 7). Currently, with the improvement of intensive care interventions, the mortality rate of extremely preterm neonates with BPD has decreased (7–9). However, many survivors still face a lifetime of disability, including long time dependence of oxygen therapy, asthma, and repeated hospital admission because of pneumonia even in developed countries (4, 6). Although new ventilation strategies and pharmacological treatments have been applied, there are no curative therapies available to target the underlying structural changes of the lungs leading to the symptoms (3).

The cord blood mononuclear cell (ACBMNC) layer is rich in valuable stem and progenitor cells (SPC) (10, 11). Although it is capable of self-renewal, it also has the potential to differentiate into various cellular phenotypes. In addition, its paracrine effect contributes to tissue repair and immune modulation (7, 11, 12). Animal studies have demonstrated the beneficial effects of the infusion of cord blood stem cells in the prevention and treatment of lung injury, including experimental BPD (13–15). Evidence from several clinical trials has proven the safety and feasibility of autologous cord blood infusion in neonates (9, 16–20). However, there is very limited data regarding its effects on preventing BPD, especially among extremely low-birth weight (ELBW) infants.

With these concepts in mind, the authors have tried to clarify the effects of ACBMNC cell infusion for the prevention of BPD since 2009. In the first study performed at our hospital, the safety and feasibility of ACBMNC infusion in preterm infants was demonstrated (9). Subsequently, we compared the effectiveness of ACBMNC infusion in those patients at our center (8). We found that ACBMNC infusion resulted in a significant decrease in the duration of mechanical ventilation (3.2 vs. 6.41 days, *p* = 0.028) and in the need for oxygen therapy (5.33 vs. 11.31 days, *p* = 0.047) (8). Given the small sample size and comparatively high gestational age of the enrolled preterm infants, no statistically significant differences in BPD incidence were observed in the previous small-sample trial. Therefore, our aim is to conduct a large-scale, multi-center, blinded randomized clinical trial (RCT) to evaluate the efficacy of ACBMNC infusion in BPD prevention in extremely preterm neonates.

## Methods and Analysis

### Study Design and Settings

**Figure 1:**
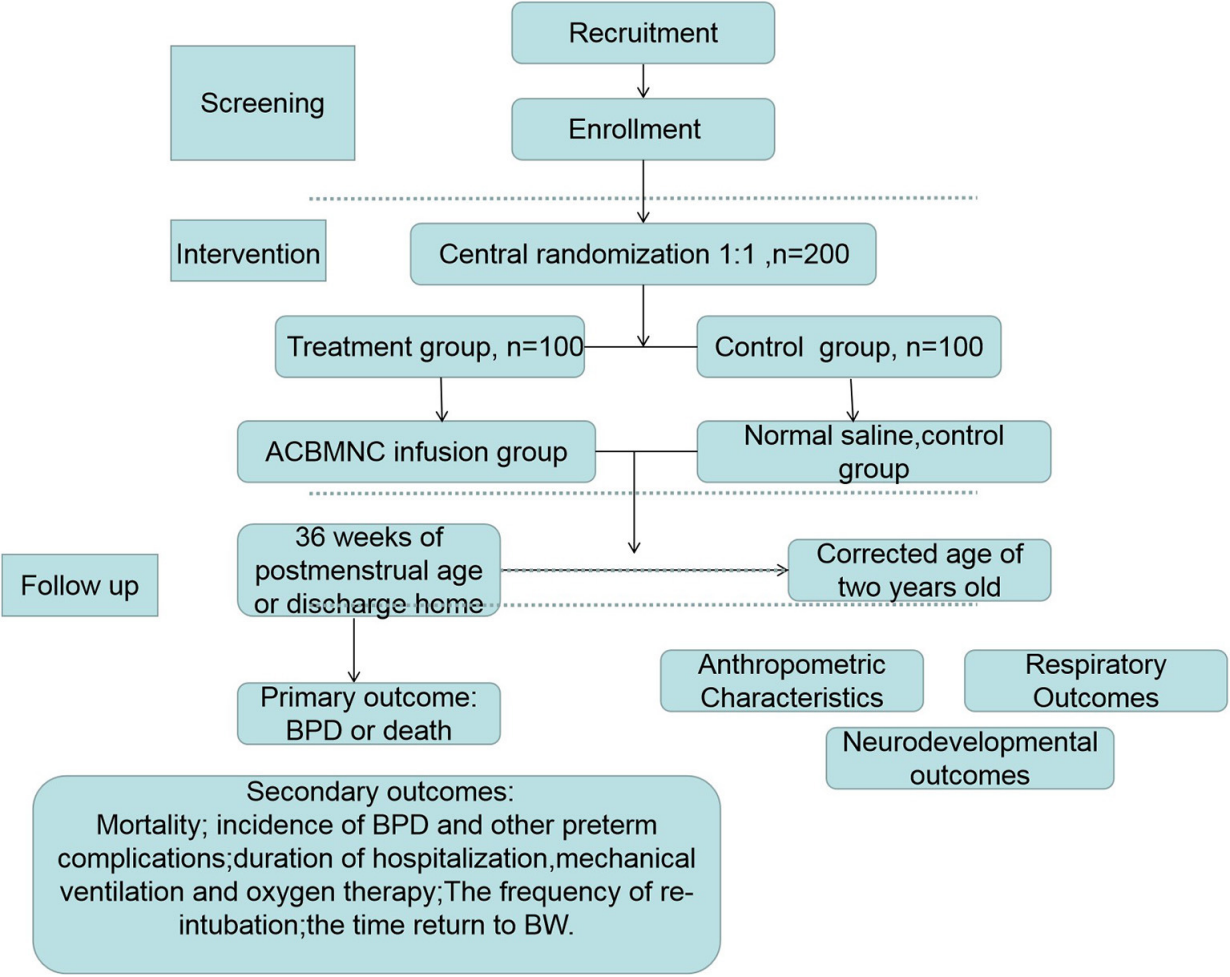
Flow diagram.

This study protocol describes a randomized, placebo-controlled, double-blinded, multi-center trial to be conducted at 14 medical centers (Table 1) in tertiary hospitals. The participating Neonatal Intensive Care Units (NICU) were selected by the expert committee based on the distance to Guang Dong Cord Blood and Stem Cell Bank and the level of intensive care that the NICUs could provide. The Guang Dong Cord Blood and Stem Cell Bank is a public provincial blood bank affiliated with the Guangdong Women and Children Hospital and collects cord blood routinely at these hospitals. To ensure that the cord blood would be processed and infused to the infants within 24 h after birth, the distance of these centers should fulfill this criterion. Furthermore, the NICUs in the selected centers are members of Guangdong Neonate Intensive Care Network. The staff working at these centers has been trained by the NICU of the Guangdong Women and Children Hospital, which means the selected centers use similar guidelines regarding treatment of patients. All the centers will hold quality control meetings frequently to ensure research consistency. A total of 200 neonates fulfilling the eligibility criteria will be enrolled. Subsequently, the participants will be randomly divided into two groups [ACBMNC infusion group and control [placebo] group] at a 1:1 ratio. The protocol for this study has been developed based on Standard Protocol Items: Recommendations for Interventional Trials (SPIRIT) checklist (Additional File 1). We have followed the Consolidated Standards of Reporting Trials (CONSORT) guidelines and the study design is illustrated in Figure 1. Consort flow diagram.

**Table 1:** Research centers.

01	Department of neonatology, Guangdong Women and Children Hospital
02	Department of neonatology, Sun Yat-sen Memorial Hospital, Sun Yat-sen University
03	Department of neonatology, The third Affiliated Hospital of Sun Yat-sen University
04	Department of neonatology, Nanfang Hospital, Southern Medical University
05	Department of neonatology, Zhongshan Boai Hospital
06	Department of neonatology, Foshan Chancheng Central Hospital
07	Department of neonatology, Foshan Women and Children Hospital
08	Department of neonatology, Guangdong Second Provincial General Hospital
09	Department of neonatology, Hexian Memorial Affiliated Hospital of Southern Medical University
10	Department of neonatology, Heyuan Women and Children Hospital
11	Department of neonatology, Jiangmen Women and Children Hospital
12	Department of neonatology, Dongguan Women and Children Hospital
13	Department of neonatology, Guangzhou Huadu Women and Children Hospital
14	Department of neonatology, The Fifth Affiliated Hospital of Guangzhou Medical University

### Trial Objectives

#### Primary Objective

The primary objective of this trial is to evaluate the efficacy of ACBMNC infusion in preventing BPD at 36 weeks of postmenstrual age or at discharge, whichever comes first, in extremely preterm infants (20).

#### Secondary Objectives

The secondary objectives of this trial are (1) to compare the infant mortality rate at 36 weeks of postmenstrual age; (2) to compare the rate of other common preterm complications included intraventricular hemorrhage (IVH), necrotizing enterocolitis (NEC), retinopathy of prematurity (ROP), respiratory distress syndrome (RDS), ventilation-associated pneumonia (VAP), hypoxic ischemic encephalopathy (HIE), late-onset sepsis (LOS), and anemia; (3) to compare the duration of mechanical ventilation and oxygen therapy in the two groups; (4) to determine re-intubation rate and time return to birth weight (BW); (5) to compare the duration of antibiotic usage; and (6) to determine the long-term outcomes after a 2-year follow up, including anthropometric characteristics, respiratory outcomes, and neurodevelopmental outcomes via standardized neurological examination.

### Participants

#### Inclusion Criteria

Infants fulfilling all the following inclusion criteria will be enrolled in this trial: (1) birth at a study hospital; (2) a singleton birth; (3) <28 weeks gestational age, (4) signed informed consent from parents before labor; and (5) available umbilical cord blood (UCB).

#### Exclusion Criteria

Infants will be excluded from the study if: (1) they exhibit severe congenital abnormalities (detected via prenatal ultrasound); (2) mothers present with clinical chorioamnionitis, and (3) mothers are positive for hepatitis B (HBsAg and/or HBeAg) or hepatitis C virus (anti-HCV), syphilis, human immunodeficiency virus (HIV) (anti-HIV-1 and−2), or IgM against cytomegalovirus (CMV), rubella, toxoplasma, and herpes simplex virus.

#### Ethical Approval

This study was approved by the ethics committee of the Guangdong Women and Children Hospital, Guangzhou Medical University.

#### Sample Size

Based on our previous study and others' study (9–16, 21), we found the ACBMNC infusion was effective in reducing respiratory support duration in preterm infants. The rate of BPD among extremely preterm infants in our NICU was 60% (pA). What we expect to be an intended (or at least acceptable) effect of the ACBMNC infusion is 25% reduction in frequency of BPD (pB:35%). To detect this difference with a sensitivity of 80% (α) and an error probability of 5% (β), at least 59 patients per randomization group will be required using the following formula:


$$\begin{array}{l} \text{n}=\left(\text{pA}\left(1-\text{pA}\right)/\text{κ}+\text{pB}\left(1-\text{pB}\right)\right)\left((\text{z}1-\alpha /2+\text{z}1-\beta )/(\text{pA}-\text{pB}\right){)}^{2} \end{array}$$

To account for the possibility of as high as 20% loss to follow-up, our estimated sample size is 140 cases totally.

#### Randomization

The randomization sequence will be generated electronically using SPSS (version 21). Following enrolment, treatment will be assigned after verification of eligibility and consent status. Computer generated randomization will be performed by the statistician in our center at a 1:1 ratio. A randomization number will be assigned by computer for each enrolled infant. Infants will be randomized to the order in which they receive ACBMNC and placebo infusions. Those enrolled in the ACBMNC group will receive an infusion of ACBMNC within 24 h after birth. Those in the placebo group will receive an infusion of a placebo solution, consisting of normal saline with the same volume. Cell dose for all patients to be targeted at 5 × 10^7^ cells per kilogram.

#### Blinding

All hospital ethics committees will review the study data during the trials. None will be involved in the study or will be aware of the treatment-group assignments for the infants. Only nurses and physicians conducting the infusions will be aware of the treatment assignment, and these individuals will have had no contact with the staff that will collect and analyze the patient data. The parents will not be aware of the treatment assignment. This study will be double-blinded.

#### Intervention

##### Cord blood processing

The Guang Dong Cord Blood and Stem Cell Bank is a public provincial blood bank accredited for stem cell manipulation by National Health Commission of the People's Republic of China and American Association of Blood Bank (AABB). Procedures for cord blood collection and processing will be performed in accordance with cord blood bank guidelines (22). The umbilical cord will be clamped for collection using a blood-collection bag (WEGO, China) containing 28 mL of citrate-phosphate-dextrose anticoagulant immediately after birth and before the placenta is delivered. The umbilical vein will be sterilized and punctured with a 17-gauge needle. UCB will be collected by trained obstetricians or cord blood bank collection staff present at the hospital during weekdays for 8–12 h per day in each center. When collection is completed, the blood bag tubing will be closed and sealed. Cord blood labeled with the full name of the donor, group type, and volume of the blood product will be stored at 4°C and sent to the Cord Blood and Stem Cell Bank for immediate processing. Before processing, 2 mL samples will be taken from all collected CB units to test for the presence of viruses (HIV, HBV, HCV, CMV) by PCR and bacterial infections via bacterial smear. A sample of peripheral blood was collected from the mother and tested for the presence of maternal transmissible diseases. The results will be obtained immediately before the start of transfusion. After a sample is taken, it will be volume- and RBC-reduced after a 30 min incubation with 6% Hespan (Bethlehem, USA) following established CBB procedures using the SEPAX S-100 automated processing system (Biosafe, Geneva, Switzerland) if the unit contained >30 mL of UCB or manually, if the unit was <30 mL. The mononuclear layer will be isolated by density gradient centrifugation (1,000 × g, 30 min, room temperature, Beckman, American), and then transferred to cryobags. Excessive nucleated cell-poor plasma will be expelled. Meanwhile, the MNC count, CD34 cell count, CFU-GM, and sterility detection (Sheldon Manufacturing Inc., Comelius, OR, USA) will be performed. Cell viability will be measured using a 7-aminoactinomycin D (7-AAD) detection kit by flow cytometry analysis (BD Bioscience, USA). After processing, the cord blood cells will be sent back to the hospitals where it was collected. All infusions will be administered at the NICU. Infusate and subject identities will be double-checked by research and clinical nursing staff. Infusions will also be monitored by research and clinical staff. Cells will be infused over 15 min, followed by a 2-mL saline flush to clear the intravascular line.

#### Trial Treatment Methods

Eligible infants will be observed at the NICU of the Guangdong Women and Children Hospital until discharge home. All patients in the study will be given intensive care therapy in accordance with departmental guidelines, which include therapies such as positive pressure mechanical ventilation, non-invasive respiratory support, oxygen therapy, and exogenous surfactant replacement (Curosurf, Chiesi, Parma, Italy). Chest radiographs will be performed at admission and 8 h after CBT on the first day of life in all surviving patients. Blood gas will be monitored every 24 h until weaning from ventilation. All clinical diagnoses will be defined according to a standard reference (16). Soon after the preterm infant is delivered, written consent will be obtained from the parents, and ACBMNC infusion was applied to the baby in addition to routine pulmonary surfactant replacement, and mechanical ventilation support as indicated. Those assigned to the ACBMNC group will receive an infusion of ACBMNC with 24 h after birth. Infants in the control group will receive an infusion of a placebo solution consisting of normal saline with the same volume. Cell dose for all patients will be targeted at 5 × 10^7^ cells per kilogram.

#### Safety Assessment

The trial will be strictly monitored by a safety monitoring board, which will be notified of specific severe adverse events (including death, LOS, NEC, severe IVH, cystic periventricular leukomalacia, and fever) within 48 h. Other adverse or unexpected events will be reviewed monthly. After each interim analysis, the data safety monitoring board will make a decision on whether to stop or continue the trial based on safety monitoring and sequential analysis of the primary outcome. Assessment of safety will be conducted at 12 and 24 h after infusion, as well as during hospitalization and return visits. Heart rate, systolic, diastolic, and mean arterial blood pressure, and arterial blood oxygen saturation levels will be monitored in the peripheral blood continually and will be documented. Moreover, laboratory investigations in the peripheral blood including blood routine tests and blood gas analysis will be monitored and kept stable during the whole treatment period. Infusion reactions and signs of circulatory overload will be checked.

#### Basic Clinical Data Collection

The following data will be collected: (1) clinical basic characteristics including sex, gestational age, birth weight, delivery mode, Apgar score at 1, 5, 10 min; (2) characteristics of cord blood processing including cord blood volume, cell number, cell concentration before and after processing, CFU-GM, CD34+ cell count, cell viability post processing; (3) characteristics of infusion cells including total cell number, time between collection (birth) and initiation of infusion, infused volume, and pathogen detection (including bacteria culture, fungus culture, HIV, HBV, HCV, CMW, and *Treponema pallidum*) results; and (4) other routine clinical interventions including dose and times of pulmonary surfactant (PS) replacement, postnatal steroid use, surgical closure of patent ductus arteriosus, and use of blood products.

We will use the following clinical definitions in this study:

1. Gestational age will be determined on the basis of a combination of the last menstrual period and early ultrasound findings
2. The diagnosis of common preterm complications will include the following (23):

BPD, defined as treatment with oxygen >21% for at least 28 days, and its severity will be assessed at 36 weeks of postmenstrual age or discharge home whichever comes first. Mild BPD, defined as breathing room air at assessment. Moderate BPD, defined as the need for <30% supplemental oxygen, and severe BPD defined as needing ≥30% supplemental oxygen or positive airway pressure. For these patients, BPD status will be analyzed by a committee of three independent experts blinded to the study group.

RDS will be defined if the infants show evidence of respiratory symptoms such as grunting and chest retraction, typical chest radiograph findings, and/or treatment with surfactant, and the need for assisted ventilation.

NEC will be defined using Bell's classification. Infants with stage II or above will be diagnosed with NEC.

LOS will be defined if the infants had a positive bacterial culture results after the first 72 h after birth.

ROP will be defined according to the International Classification for Retinopathy of Prematurity.

Anemia will be defined as hemoglobin no more than 140 g/L.

IVH and periventricular leukomalacia (PVL) will be defined by serial head ultrasound, performed according to the description by Volpe. The first head ultrasound will be performed within 3 days after birth and follow-up head ultrasound examinations will be performed every week until the day of discharge.

VAP will be defined as a pneumonia occurring after the patient has been intubated and has received mechanical ventilation for more than 48 h.

### Outcome Measures

*Primary outcome measure:*

- The frequency of BPD or death in at 36 weeks of postmenstrual age or discharge home whichever comes first.

*Secondary outcome measures:*

- Mortality rate
- Incidence of BPD
- Incidence of other preterm complications including IVH, PVL, NEC, ROP, RDS, VAP, LOS, and anemia [all clinical diagnoses will be defined according to a standard references (16)].
- Duration of hospitalization.
- Duration of mechanical ventilation and oxygen therapy
- Frequency of re-intubation.
- The time (days) return to BW.

#### Follow Up

All the infants will be followed until the corrected age of 2 years old, the following parameters will be recorded.

- Anthropometric characteristics: weight, length, and head circumference.
- Respiratory outcomes including the occurrence of wheezing, asthma, nocturnal cough, supplemental oxygen requirement, and rehospitalization because of pneumonia.
- Degree of neurodevelopmental impairment via standardized neurological examination [using the revised Brunet-Lézine [RBL] global developmental quotient score (16)], and other major neurodevelopmental outcomes including cerebral palsy, seizures, auditory impairment, and visual impairment (Table 2).

**Table 2:** Contents and points of data capture: Standard Protocol Items: Recommendations for Interventional Trials (SPIRIT) schedule of enrolment, interventions, and assessments.

**Visit**	**Screening**	**Intervention**	**Follow up**	
	**V1**	**V2**	**V3**	**V4**
Time point	born	Within 24 h after birth	36 weeks of postmenstrual age	Corrected age of 2 years old
Informed consent form	√			
Screening the subject	√			
Demographic information	√			
Inclusion/exclusion criteria	√			
Get random number	√			
Cord blood process		√		
Vital signs	√	√	√	
Chest radiographs		√		
Pulmonary surfactant replacement		√		
Mechanical ventilation	√	√		
Arterial blood oxygen saturation	√	√		
Laboratory tests	√	√	√	
Blood routine test	√	√		
Blood gas	√	√		
Safety outcomes		√	√	√
Record adverse events		√	√	√
Postnatal steroid use		√	√	√
Surgical closure of patent ductus arteriosus		√	√	
Blood products use		√	√	
Bronchopulmonary dysplasia			√	
Other preterm complications			√	
Duration of hospitalization			√	√
Anthropometric Characteristics			√	√
Respiratory Outcomes				√
Neurodevelopmental outcomes				√

### Statistical Analysis

Statistical analyses between the two groups will be performed using an unpaired two-tailed Student's *t*-test or Chi-squared Test as appropriate. A logistic regression model will be used for the entire study population (i.e., without removing deaths) to adjust for the effect of treatment on primary outcome according to baseline characteristics and events known to affect the occurrence of BPD (i.e., gestational age, birth weight and other characters that are different in two groups after initial analysis), and to investigate the effect of treatment on the following outcomes: extubation rates, severe adverse events, and death before discharge. The Breslow-Day test and Corchran-Mental-Haenszel (CMH) test will be used to adjust the data derived from different participating centers. The results will be reported as odds ratio (OR) with 95% confidence intervals (CI). All statistical tests will be two-tailed and a *p* < 0.05 will be considered statistically significant. All statistical analysis will be performed using SPSS 21.0 (IBM).

## Discussion

BPD is still a major complication of prematurity. Currently, therapy for BPD includes non-invasive ventilation strategies, inhaled nitric oxide, antioxidants, vitamin A, caffeine, and corticosteroids, however, these strategies are mainly palliative and do not address the underlying structural changes involved in BPD including the reduced numbers of alveoli, blood vessels, and prominent fibrosis of the lungs (24–29). Stem cells are showed to have beneficial effects on both treatment and prevention of BPD in several preclinical and clinical settings (16–19, 30–34). Cord blood MNCs are rich in stem cells. As a convenient and safe source of stem cells, it could provide regrowth to underdeveloped lung tissue of preterm infants by providing more stem/progenitor cells. In addition, its paracrine effects help to improve lung function, vascularization of the airways, and reduce fibrosis, and therefore have considerable potential for reducing lung injury in preterm infants (35–40). However, few studies have evaluated the effects of ACBMNC infusion for prevention of BPD in extremely preterm infants.

The outcome of our study would be a major step forward solving this problem. To our knowledge, this would be the first clinical trial to assess ACBMNC infusion soon after birth in extremely preterm neonates in terms of the rate of BPD and other prematurity-related complications. Considering the novelty of the therapy and the randomized double-blinded characters of this study, it may take longer time to complete this trial. These may be potential shortcomings of the study.

## Conclusion

In conclusion, this randomized, placebo controlled, double blinded study aims to investigate the effects of ACBMNC for preventing BPD in extremely preterm infants.

## Ethics Statement

The studies involving human participants were reviewed and approved by [Guangdong Women and Children Hospital Ethics committee]. The legal guardian of participants provided written informed consent to participate in this study.

## Author Contributions

All authors read and approved the final manuscript.

### Conflict of Interest

The authors declare that the research was conducted in the absence of any commercial or financial relationships that could be construed as a potential conflict of interest.
