# Corrigendum: Use of autologous cord blood mononuclear cells infusion for the prevention of bronchopulmonary dysplasia in extremely preterm neonates: a study protocol for a placebo-controlled randomized multicenter trial [NCT03053076]

**DOI:** 10.3389/fped.2023.1284824

**Published:** 2023-10-11

**Authors:** Zhuxiao Ren, Xu Fang, Qi Zhang, Y. G. Mai, X. Y. Tang, Q. Q. Wang, C. H. Lai, W. H. Mo, Y. H. Dai, Q. Meng, Jing Wu, Z. Z. Ao, H. Q. Jiang, Yong Yang, L. H. Qu, C. B. Deng, Wei Wei, Yongsheng Li, QI Wang, Jie Yang

**Affiliations:** ^1^ Department of Neonatology, School of Medicine, Jinan University, Guangzhou, China; ^2^ Department of Neonatology, Guangdong Women and Children Hospital, Guangzhou Medical University, Guangzhou, China; ^3^ Department of Neonatology, Sun Yat-sen Memorial Hospital, Sun Yat-sen University, Guangzhou, China; ^4^ Department of Neonatology, The Third Affiliated Hospital of Sun Yat-sen University, Guangzhou, China; ^5^ Department of Neonatology, Nanfang Hospital, Southern Medical University, Guangzhou, China; ^6^ Department of Neonatology, Zhongshan Boai Hospital, Zhongshan, China; ^7^ Department of Neonatology, Foshan Chancheng Central Hospital, Foshan, China; ^8^ Department of Neonatology, Foshan Women and Children Hospital, Foshan, China; ^9^ Department of Neonatology, Guangdong Second Provincial General Hospital, Guangzhou, China; ^10^ Department of Neonatology, Hexian Memorial Affiliated Hospital of Southern Medical University, Guangzhou, China; ^11^ Department of Neonatology, Heyuan Women and Children Hospital, Heyuan, China; ^12^ Department of Neonatology, Jiangmen Women and Children Hospital, Jiangmen, China; ^13^ Department of Neonatology, Dongguan Women and Children Hospital, Dongguan, China; ^14^ Department of Neonatology, Guangzhou Huadu Women and Children Hospital, Guangzhou, China; ^15^ Department of Neonatology, The Fifth Affiliated Hospital of Guangzhou Medical University, Guangzhou, China; ^16^ Guang Dong Cord Blood and Stem Cell Bank, Guangzhou, China

**Keywords:** crod blood cells, bronchopulmonary dysplasia, extremely preterm neonates, prevention, autologous

A Corrigendum on Use of autologous cord blood mononuclear cells infusion for the prevention of bronchopulmonary dysplasia in extremely preterm neonates: a study protocol for a placebo-controlled randomized multicenter trial [NCT04440670] By Ren Z, Fang X, Zhang Q, Mai YG, Tang XY, Wang QQ, Lai CH, Mo WH, Dai YH, Meng Q, Wu J, Ao ZZ, Jiang HQ, Yang Y, Qu LH, Deng CB, Wei W, Li Y, Wang Q and Yang J. (2020) Front. Pediatr. 8:136. doi: 10.3389/fped.2020.00136

## Error in the article title

In the published article, there was an error in the trial number in the title. The title “Use of Autologous Cord Blood Mononuclear Cells Infusion for the Prevention of Bronchopulmonary Dysplasia in Extremely Preterm Neonates: A Study Protocol for a Placebo-Controlled Randomized Multicenter Trial [NCT03053076]” has been changed to “Use of Autologous Cord Blood Mononuclear Cells Infusion for the Prevention of Bronchopulmonary Dysplasia in Extremely Preterm Neonates: A Study Protocol for a Placebo-Controlled Randomized Multicenter Trial [NCT04440670]”.

## Text correction

In the published article the Clinical Trial Registration information was not correct. A correction has been made to **Abstract** section, **Clinical Trial Registration** subsection.

This sentence previously stated:

“Clinical Trial registration: ClinicalTrials.gov, NCT03053076, registered 02/14/2017, retrospectively registered, https://register.clinicaltrials.gov/prs/app/action/SelectProtocol?sid = S0006WN4&selectaction = Edit&uid = U0002PLA&ts = 2&cx = 9y23d4”

The corrected sentence appears below:

“Clinical Trial registration: ClinicalTrials.gov, NCT04440670, registered 06/18/2020, prospectively registered, https://clinicaltrials.gov/study/NCT04440670?term = NCT04440670&rank = 1”

Furthermore, the sample size calculation number was not correct. A correction has been made to **Methods and Analysis** section, **Participants** subsection, **Sample size** paragraph.

This sentence previously stated:

“Based on our previous study and others’ study, we found the ACBMNC infusion was effective in reducing respiratory support duration in preterm infants. The rate of BPD among extremely preterm infants in our NICU was 60% (p1). What we expect to be an intended (or at least acceptable) effect of the ACBMNC infusion is 25% reduction in (p2) frequency of BPD. To detect this difference with a sensitivity of 80% and an error probability of 5%, at least 94 patients per randomization group will be required using the following formula:

$$n\;=\;(\mathrm{pA}(1-\mathrm{pA})\kappa +\mathrm{pB}(1-\mathrm{pB}))(z1-\alpha /2\;+\;z1-\beta \mathrm{pA}-\mathrm{pB}{)}^{2}$$

To account for the possibility of loss to follow-up, our estimated sample size is 200 cases.”

The corrected sentence appears below:

“Based on our previous study and others’ study, we found the ACBMNC infusion was effective in reducing respiratory support duration in preterm infants. The rate of BPD among extremely preterm infants in our NICU was 60% (pA). What we expect to be an intended (or at least acceptable) effect of the ACBMNC infusion is 25% reduction in frequency of BPD (pB: 35%). To detect this difference with a sensitivity of 80% and an error probability of 5%, at least 59 patients per randomization group will be required using the following formula:

$$\begin{array}{r} n=(\mathrm{pA}(1-\mathrm{pA})/\kappa +\mathrm{pB}(1-\mathrm{pB})) \end{array}(\left(z1-\alpha /2+z1-\beta )/(\mathrm{pA}-\mathrm{pB}\right){)}^{2}$$

To account for the possibility of as high as 20% loss to follow-up, our estimated sample size is 140 cases totally.”

The authors apologize for these errors and state that this does not change the scientific conclusions of the article in any way.

