# Supplementary material for: Use of Autologous Cord Blood Mononuclear Cells Infusion for the Prevention of Bronchopulmonary Dysplasia in Extremely Preterm Neonates: A Study Protocol for a Placebo-Controlled Randomized Multicenter Trial [NCT04440670]
Source: Front Pediatr. 2020 Apr 2;8:136. doi: 10.3389/fped.2020.00136 (PMC7142259; doi:10.3389/fped.2020.00136)
Supplement: Supplementary file 2 [file Data_Sheet_2.doc]

Additional file 2 World Health Organization Trial Registration Data Set

| **Item** | **Description** |
| --- | --- |
| 1. Primary registry and trial-identifying number | ClinicalTrials.gov ID:**NCT03053076** |
| 2. Date of registration in primary registry | 14 February 2017 |
| 3. Secondary identifying numbers | Not applicable,NA |
| 4. Sources of monetary or material support | Guang Dong Cord Blood and Stem Cell Bank, Guangzhou , China |
| 5. Primary sponsor | Guang Dong Cord Blood and Stem Cell Bank, Guangzhou , China |
| 6. Secondary sponsor(s) | NA |
| 7. Contact for public queries | Yang Jie , jieyang0830@126.com |
| 8. Contact for scientific queries | Yang Jie , jieyang0830@126.com |
| 9. Public title | The efficacy and safety of Xuebijing injection for acute exacerbations of chronic obstructive pulmonary disease |
| 10. Scientific title | **Effect of Autologous cord blood mononuclear cells for prevention of bronchopulmonary dysplasia in extremely preterm neonates: design and rationale of A placebo-controlled randomized multicenter trial** |
| 11. Countries of recruitment | China |
| 12. Health condition(s) or problem(s) studied | **bronchopulmonary dysplasia in extremely preterm neonates** |
| 13. Intervention(s) | The treatment group: autologous cord blood mononuclear cells infusion (5×10^7^cells/kg)  The control group: normal saline (dosage: 8ml).  Within 24h after birth |
| 14. Key inclusion and exclusion criteria | Inclusion criteria:   1. born at study hospital; 2. singleton birth; 3. less than 28 weeks GA 4. Signed informed consent obtained; 5. had available umbilical cord blood (UCB).   Exclusion criteria:   1. with severe congenital abnormalities; 2. with maternal clinical chorioamnionitis   3. the mother was positive for hepatitis B (HBsAg and/or HBeAg) or C virus (anti-HCV), syphilis, HIV (anti-HIV-1 and -2) or IgM against cytomegalovirus, rubella, toxoplasma and herpes simplex virus. |
| 15. Study type | Randomized, blinded, controlled trial |
| 16. Date of first enrollment | 1^st^ October 2019 |
| 17. Target sample size | 200 |
| 18. Recruitment status | Recruiting |
| 19. Primary outcome(s) | Survival without BPD |
| 20. Key secondary outcome(s) | -To compare the mortality rate at 36 weeks of postmenstrual age  -To compare the rate of other common preterm complications included intraventricular hemorrhage (IVH), necrotizing enterocolitis (NEC), retinopathy of prematurity (ROP), respiratory distress syndrome (RDS), ventilation-associated pneumonia (VAP), hypoxic ischemic encephalopathy (HIE), late onset sepsis (LOS) and anemia.  -To compare the duration of mechanical ventilation and oxygen therapy in two groups  - To determine re-intubation rate and time return to BW  - To compare the duration of antibiotic usage  - To determine the long term outcomes after two years follow up |
